# A study protocol of a randomised controlled trial incorporating a health economic analysis to investigate if additional allied health services for rehabilitation reduce length of stay without compromising patient outcomes

**DOI:** 10.1186/1472-6963-10-308

**Published:** 2010-11-12

**Authors:** Nicholas F Taylor, Natasha K Brusco, Jennifer J Watts, Nora Shields, Casey Peiris, Natalie Sullivan, Genevieve Kennedy, Cheng Kwong Teo, Allison Farley, Kylee Lockwood, Camilla Radia-George

**Affiliations:** 1 Eastern Health, 5 Arnold Street, Box Hill, Victoria, 3128. Australia; 2 School of Physiotherapy and Musculoskeletal Research Centre, La Trobe University, Victoria, 3086. Australia; 3 Centre for Health Economics, Monash University, Clayton, Victoria, 3800. Australia; 4 Cabrini Health, 183 Wattletree Road, Malvern, Victoria, 3144. Australia

## Abstract

**Background:**

Reducing patient length of stay is a high priority for health service providers. Preliminary information suggests additional Saturday rehabilitation services could reduce the time a patient stays in hospital by three days. This large trial will examine if providing additional physiotherapy and occupational therapy services on a Saturday reduces health care costs, and improves the health of hospital inpatients receiving rehabilitation compared to the usual Monday to Friday service. We will also investigate the cost effectiveness and patient outcomes of such a service.

**Methods/Design:**

A randomised controlled trial will evaluate the effect of providing additional physiotherapy and occupational therapy for rehabilitation. Seven hundred and twelve patients receiving inpatient rehabilitation at two metropolitan sites will be randomly allocated to the intervention group or control group. The control group will receive usual care physiotherapy and occupational therapy from Monday to Friday while the intervention group will receive the same amount of rehabilitation as the control group Monday to Friday plus a full physiotherapy and occupational therapy service on Saturday. The primary outcomes will be patient length of stay, quality of life (EuroQol questionnaire), the Functional Independence Measure (FIM), and health utilization and cost data. Secondary outcomes will assess clinical outcomes relevant to the goals of therapy: the 10 metre walk test, the timed up and go test, the Personal Care Participation Assessment and Resource Tool (PC PART), and the modified motor assessment scale. Blinded assessors will assess outcomes at admission and discharge, and follow up data on quality of life, function and health care costs will be collected at 6 and 12 months after discharge. Between group differences will be analysed with analysis of covariance using baseline measures as the covariate. A health economic analysis will be carried out alongside the randomised controlled trial.

**Discussion:**

This paper outlines the study protocol for the first fully powered randomised controlled trial incorporating a health economic analysis to establish if additional Saturday allied health services for rehabilitation inpatients reduces length of stay without compromising discharge outcomes. If successful, this trial will have substantial health benefits for the patients and for organizations delivering rehabilitation services.

**Clinical trial registration number:**

Australian and New Zealand Clinical Trials Registry ACTRN12609000973213

## Background

Reducing patient length of stay is a high priority for health service providers and is considered to be an indicator of efficiency [1,2]. The implications of a reduction in length of stay are potentially significant for the individual, the health service and for the community. A reduction in length of stay means the individual can return to their community sooner, that individuals may not have to wait so long for a bed, that the health service can treat more patients, and that there are cost savings for the community. However, from the perspective of the health system, if reducing length of stay is achieved at the expense of quality of care, this will reduce efficiency and could increase pressures and costs to other health services. Despite this concern, there is some evidence, at least in acute hospitals, that reducing length of stay has not been achieved at the expense of quality of care [3,4].

The allied health team, including physiotherapists and occupational therapists, play a key role in the effective management of patients receiving rehabilitation for neurological and musculoskeletal conditions [5-10]. Physiotherapy and occupational therapy aim to increase the independence of patients receiving rehabilitation by improving mobility and self-care skills. Developing independent mobility and self care skills are important physical factors in predicting the need for long-term placement [11] and are associated with better quality of life [12]. Therefore, attaining mobility and self-care skills are important in determining whether patients receiving rehabilitation can successfully return home to participate in their usual societal activities and improve their quality of life.

There has been an increased focus on the importance of intensity and activity in rehabilitation. During early rehabilitation, patients receive relatively little therapy and have low levels of physical activity [13]. Reports that increasing the intensity of rehabilitation could be associated with improved outcomes [14] have led to calls for rehabilitation to be more active and intensive [15,16]. Previous studies have indicated that the intensity of physiotherapy and occupational therapy provision may affect patient outcomes leading to reduced mortality following stroke [17], and improved functional status following brain injury [18], stroke [17,19], and femoral fracture [20].

One way to increase the intensity of rehabilitation is to provide additional allied health services. Typically, allied health rehabilitation services are provided Monday to Friday. Weekend services have not been provided, have been provided on a much reduced scale, or have only been provided to patients at risk of an acute event such as due to a chest infection [21-24]. A recent systematic review provided preliminary evidence that additional allied health services can reduce length of stay in hospital for patients receiving rehabilitation [25]. There was evidence that additional weekend physiotherapy significantly decreased length of stay for patients who had undergone elective hip and knee joint arthroplasty [26] and patients with non-surgical stroke [27]. However, trial quality in the systematic review was only fair, with only three of nine included studies using a randomised control design.

Our pilot study examined the effect of additional Saturday physiotherapy for rehabilitation inpatients [28]. Two hundred and sixty two participants were included in this randomised controlled trial. The mean patient length of stay was 24.4 days (SD 15.9) for patients who received a Monday to Friday physiotherapy service and 21.2 days (SD 14.0) for patients who received a Monday to Saturday service. The 3.2 day difference demonstrated a trend in favour of the intervention group (95% CI -0.5 to 6.9 days, *p*= 0.09). Assuming a maintained effect size of 0.21 and power of 0.80, then 356 participants would have been required in each group to reach statistical significance.

Patient quality of care was not adversely affected by a shortened hospital stay [28]. There were no differences in function, quality of life and discharge outcomes between the Monday to Friday service and the Monday to Saturday service. Quality of life was not significantly different between the groups at discharge or at follow-up. There was no significant difference between groups in the change from admission to discharge for the secondary outcome measures of functional independence measure, timed 10 metre walk test, timed up and go, and the modified motor assessment scale. There were also no significant differences in discharge destination, adverse events or follow-up therapy between the groups.

In a healthcare system with fixed capacity even a small saving in length of stay can be important. The Australasian College for Emergency Medicine recently reported that if a hospital with 20,000 admissions each year saved half a bed day per patient by improving discharges, about 2000 extra patients a year could be admitted [29]. Data from our pilot study suggests that if the average length of stay from each rehabilitation episode was reduced by 3.2 days, there would be an additional 68 patients admitted to a typical 30-bed rehabilitation facility each year [28]. These results have the potential to increase patient flow from the acute to the subacute setting providing increased capacity in the acute care setting. A rigorous health economic evaluation in a larger trial is required to confirm the cost effectiveness of providing additional allied health services for rehabilitation.

In summary, to evaluate the potential benefits of the provision of additional Saturday allied health services to rehabilitation inpatients, we will conduct a fully powered randomized controlled clinical trial that will: complete an economic analysis of additional allied health services compared with the traditional Monday to Friday usual care service from a healthcare system perspective; and incorporate occupational therapy rehabilitation services in addition to physiotherapy.

The primary aim of this trial is to determine if the provision of additional Saturday allied health services will reduce the costs of care by reducing patient rehabilitation length of stay without affecting health related quality of life and functional outcomes compared to those receiving usual care. The secondary aims are to examine if the provision of additional Saturday allied health services has any effect on therapeutic functional goals at discharge; or the use of community-based health services post discharge from the sub-acute hospital setting, compared to those receiving usual care. For the purpose of this clinical trial the term "allied health" will specifically refer to physiotherapy and occupational therapy services.

## Methods/Design

### Research design

The research design is a single blinded randomised controlled trial of additional Saturday allied health services for rehabilitation inpatients compared with the comparison group receiving a Monday to Friday usual care allied health service. We will conduct a clinical trial, incorporating an economic analysis, across two rehabilitation facilities with approximately 90 rehabilitation beds (providing services to eastern metropolitan Melbourne, Australia). Participants in the comparison group will receive five days of allied health services (Monday to Friday) and participants in the intervention group will receive six days of allied health services (Monday to Saturday). The trial has received ethics approval from Eastern Health Ethics Committee and La Trobe University Faculty Human Ethics Committee.

### Participants

Participants will be included if they are:

• aged 18 years or older

• have been admitted for rehabilitation at either of the two rehabilitation facilities.

Participants will be excluded if they

• do not give informed consent to participate in the trial, or

• if they are admitted for geriatric evaluation and management, as this patient group are managed differently to patients admitted for rehabilitation, or

• if they are already enrolled in another intervention trial.

Participants will not be excluded if their primary language is a language other than English. A language interpreter will be provided to ensure these patients understand the informed consent procedure and to assist them with the administration of the quality of life and clinical outcome measures. Participants will not be excluded if assessment indicates reduced cognition, as indicated by a score of less than 24/30 on the Mini-mental State Examination [30] since cognitive impairment is common in patients receiving rehabilitation [31]. Our pilot data found that 24% of patients admitted for rehabilitation had severe cognitive impairment [28]. Written informed consent will be sought from all eligible patients admitted for rehabilitation. For patients with impaired cognition or aphasia, the next of kin will be approached for informed consent.

### Randomisation

Participants will be randomised to the additional allied health services (intervention) group or the control group with a concealed method, using permuted blocks of 4, 6 and 8, stratified for case-mix status (neurological and other/orthopaedic) and site (two sites). Stratification will include site in order to facilitate the staffing of the Saturday service. The block allocation sequence will be generated by http://www.randomization.com and assignments sealed in sequentially numbered opaque envelopes. Only after the participant has enrolled in the trial and completed written informed consent and baseline testing will assignment be made by the project officer by opening the next envelope in the sequence. A member of the research team who is not involved in participant recruitment, assessment or treatment will be responsible for preparing the envelopes.

### Intervention

Usual care physiotherapy and occupational therapy will be provided to participants in the control group daily from Monday to Friday. The specific intervention will be at the discretion of the treating therapists.

The intervention group will receive the same amount of intervention as the control group Monday to Friday, plus a full service on Saturday, equating to an additional one hour of physiotherapy and one hour of occupational therapy. The type of intervention provided at the weekend will be decided by the patient's regular physiotherapist and occupational therapist, and instructions will be provided via a written handover to the weekend therapist administering the additional treatment. Provision will be made to ensure that if a participant in the control group required urgent physiotherapy at the weekend that it would be provided as normal, for example if they developed an acute respiratory complication requiring cardiorespiratory physiotherapy.

### Outcome measures

Blinded assessors will complete the baseline, discharge and follow-up assessments. The success of blinding will be evaluated at the final assessment by asking assessors to estimate their patient group allocation. During the pilot trial assessors correctly guessed group allocation of the intervention group in 61% of cases and of the control group in 64% of cases [28].

### Primary outcomes measures

Patient length of stay will be measured as the number of overnight stays in the rehabilitation unit, from the day of admission until the day of discharge from the unit. Length of stay is the largest contributor of direct care costs during rehabilitation [32,33].

The EuroQol questionnaire (EQ-5D) and the EQ-VAS [34] will be used to measure the participant's quality of life, covering the domains of mobility, self-care, usual activities, pain/discomfort, and anxiety/depression, and overall health state (EQ-VAS). The EQ-5 D is a standardised instrument for measuring health related quality of life and providing a single index of utility, necessary for the calculation of a cost utility ratio in an economic analysis. It has been used for a range of conditions and changes in the EQ-5 D are significantly correlated with changes in condition-specific measures over three months (*p*= 0.01) [35].

The Functional Independence Measure (FIM) [36] will be used to assess the amount of assistance required to complete activities including feeding, dressing, transfers, walking, stairs, and cognitive function. The FIM has demonstrated strong psychometric qualities in rehabilitation settings with high levels of reliability (ICC = .96) [37] and evidence for responsiveness and validity as a global disability measure for patients receiving rehabilitation [38].

Follow-up measures of the FIM and EuroQol at 6 and 12 months will be collected by telephone by assessors blinded to group allocation. There is evidence that telephone administration is a valid method of assessing functional status in older patients after rehabilitation [39].

Health utilisation and cost data will be collected from the rehabilitation facilities (inpatient care), outpatient services, and via a patient health service utilisation questionnaire administered at 6 months and 12 months post intervention. The questionnaire will include questions on health service utilisation including allied health and medical services, pharmaceutical use, and hospital admissions. In addition, Medicare Australia claims records will be retrieved with patient permission for 12 months post discharge to determine health service use post discharge including allied health, medical services, pharmaceutical use and hospital admissions. The amount of physiotherapy and occupational therapy intervention (number of treatment sessions, discharge planning, equipment hire or purchase, team meetings, staff discussions and family meetings) received by each group will be recorded via each hospital's allied health access database program, including the additional Saturday interventions received by the intervention group and any missed sessions as a result of medical instability, patient refusal and a patient not being ready to attend treatment on time.

The use of these tools will enable us to complete a cost utility analysis examining the cost per quality adjusted life-year saved and cost per 10% change in functional independence. We will assess health related quality of life and functional status at both 6 months and 12 months to try and minimise loss-to-follow-up as an intention-to-treat analysis will be undertaken.

### Secondary outcome measures

Secondary outcomes measures include the 10 metre walk test [40], the timed up and go test [41], and the Personal Care Participation Assessment and Resource Tool (PC-PART) [42]. The modified motor assessment scale [43] will be completed on patients with a neurological diagnosis. All secondary outcome measures are designed to evaluate clinical outcomes relevant to the functional goals of therapy, and will be measured on admission and at discharge.

Discharge destination will be recorded as home, high level residential care, low level residential care, and acute hospital transfer. This will be recorded as "worse" when the participant was discharged to a destination where more assistance would be provided, for example a high level residential care facility, or "same" when a participant was discharged to the same place of accommodation prior to the hospital admission. This outcome measure is included to measure any increase in burden of care to the community.

Demographic data on participant age, gender and co-morbidities will be documented from each participant's medical chart. Co-morbidities will be recorded using the Charlson co-morbidity index [44].

Adverse events, including falls, nosocomial infections and mortality will be recorded on an incident report form through an audit of participants at discharge and the rehabilitation facilities' incident reporting database. Follow-up physiotherapy and occupational therapy will be recorded at discharge as outpatient, community rehabilitation (including centre-based and home services), or no follow up.

## Data analysis

### Sample size estimation

Sample size estimation is based on rehabilitation length of stay data [28]. For length of stay, with a maintained effect size of .21 and power of 0.80 a sample size of 356 participants in each group is required (total of 712 participants).

### Statistical analysis

Between group differences of the primary outcomes (length of stay, EuroQol, and FIM) will be analysed with analysis of covariance of discharge scores with the baselines score as covariate [45]. Intention to treat analysis will be used with any missing health outcome data imputed using the last value carry forward method [46].

A rigorous economic evaluation will be carried out alongside the randomised clinical trial. Cost effectiveness of the intervention will be determined as the incremental cost of additional Saturday allied health inpatient rehabilitation services compared to usual inpatient rehabilitation per quality adjusted life year saved. The primary measure of incremental effect for the economic evaluation will include the difference in health related quality of life between the control and treatment groups using the EuroQol. A secondary incremental cost effectiveness ratio will be determined using the cost per change in functional status using the FIM.

Patient level costing and utilisation data for inpatient rehabilitation services from the hospital's costing and admission and discharge system will be used to estimate the costs of inpatient care. Following discharge, the patient health service utilisation questionnaire will be administered at 6 and 12 months to determine the health care services used post discharge. Costs will be attributed to these services based on published costing data from the National Hospital Cost Data Collection, Medicare Australia, and the Pharmaceutical Benefits Scheme. Where a patient is re-admitted to one of the participating rehabilitation facilities within the 12-month period inpatient costing data will be used. Medicare Australia data will be used to obtain private health service utilisation. The costs of implementing the intervention (Monday to Saturday rehabilitation) and the control programs (Monday to Friday rehabilitation) will be determined from the rehabilitation facility. This will include therapist time, equipment, consumables and administrative overheads. The incremental cost estimate will be determined as the difference in cost of implementing the Saturday allied health rehabilitation service plus the difference in health service costs (including out-of-pocket private expenditure) used in the 12 months post discharge between the control and intervention groups. A secondary incremental cost estimated will be calculated as the change in resource use resulting from the additional Saturday allied health rehabilitation service compared to usual Monday to Friday rehabilitation at the point of discharge from the inpatient rehabilitation facility. Any missing health service utilisation or cost data, where service use was recorded but a quantity not specified, will be imputed using within group mean cost [47].

The primary health economic analysis is a cost utility analysis from a health care system perspective, with outcomes based on health related quality of life (using EuroQol), and cost effectiveness using an intermediate clinical outcome (based on the FIM). We will be undertaking an intention to treat analysis where the cost utility ratio will be calculated as the change in total program and health service cost per change in quality adjusted life year saved in the intervention and control groups at 6 and 12 months. Additional cost effectiveness ratios will be calculated as the total program cost per 10% change in functional independence (at discharge), and the total program and health service cost per 10% change in functional status between the control and intervention groups over a 12 month period. One way sensitivity analysis will be undertaken to investigate the robustness of the cost effectiveness ratio to a range of cost and effect estimates, including alternative delivery arrangements, wage rates and program length on the cost side, and health related quality of life and FIM on the effect side.

Between group differences of secondary outcomes measured on continuous or ordinal scales will be analysed with analysis of covariance of discharge scores with the baselines score as covariate [45]. Between-group differences in discharge destination (home versus other) will be analysed with relative risk ratios.

## Discussion

This paper outlines the study protocol for the first fully powered prospective randomised clinical trial to establish if additional Saturday allied health services to rehabilitation inpatients reduces length of stay without compromising discharge outcomes. An economic analysis will determine the cost effectiveness of providing additional allied health services.

If successful this project will have substantial health benefits for the patient, the health care system, and for the organisation delivering health services, especially in the sub-acute sector. Increasing capacity in the sub-acute sector will have considerable flow-on effects into the acute health care sector. The results of this project will be applicable to the more than 18,000 patients receiving episodes of rehabilitation care in the public health system in Australia each year [48]. Based on the average length of stay in rehabilitation in the public system in Australia of 26.7 days [48], reductions of length of stay observed in our pilot project of about 3 days if confirmed in the proposed trial have the potential to save more than $40 M, and would allow more than 2,200 extra patients to receive rehabilitation each year across Australia. Our economic evaluation analysis will allow a rigorous evaluation of the potential benefits of providing additional rehabilitation and include health care costs other than length of stay and take into account functional outcomes and quality of life after discharge.

## List of abbreviations

CI: confidence interval; EQ-5D: EuroQol questionnaire; EQ-VAS: EuroQol visual analogue scale; FIM: Functional Independence Measure; ICC: Intraclass Correlation Coefficient; PC-PART: Personal Care Participation Assessment and Resource Tool; SD: Standard deviation

## Competing interests

The authors declare that they have no competing interests.

## Authors' contributions

NFT, NB and NoS reviewed the literature, designed the study and drafted the manuscript. JJW designed the health economic evaluation and contributed to the writing of the paper by revising it critically for important intellectual content. NaS, GK, CKT, KL, AF, CP and CRG all contributed to study design, and contributed to the writing of the paper by revising it critically for important intellectual content. All authors read and approved the final manuscript.

## Pre-publication history

The pre-publication history for this paper can be accessed here:

http://www.biomedcentral.com/1472-6963/10/308/prepub
